# Exploring the link between exposure to sexual interpersonal violence and subsequent child sexual offending: Insights from a Danish Study

**DOI:** 10.1192/j.eurpsy.2025.805

**Published:** 2025-08-26

**Authors:** F. M. Monshizadeh Tehrani, E. Kristensen, A. Giraldi, S. Bengtson

**Affiliations:** 1 Child and Adolescent Mental Health Center, Mental Health Services in the Capital Region of Denmark, Copenhagen, Denmark; 2 Forensic Psychiatry Working Group, European Federation of Psychiatric Trainees, Brussels, Belgium; 3 Sexological Clinic, Mental Health Services in the Capital Region of Denmark; 4 Department of Clinical Medicine, University of Copenhagen, Copenhagen, Denmark

## Abstract

**Introduction:**

Child sexual offending is a significant societal concern with profound consequences. While some individuals with a history of childhood sexual abuse (CSA) may reenact their traumatic experiences later in life, the link between CSA and subsequent offending patterns among individuals convicted of child sexual offending (ICSOs) remains complex and under-researched.

**Objectives:**

This study aimed to investigate the prevalence of self-reported physical and sexual violence among ICSOs and explore the link between self-experienced CSA and offending behavior, particularly focusing on the age and relationship with their victims. We hypothesized that ICSOs would replicate their own victimization experiences when perpetrating CSA.

**Methods:**

A cohort of 78 male ICSOs referred to the Danish Sexual Offender Treatment and Research Program (DASOP) between October 1, 1997, and October 1, 2001, for court-ordered pre/post-trial evaluations was analyzed. Data on self-reported experiences of CSA, physical violence, and characteristics of their victims were collected and examined for patterns.

**Results:**

Of the 31% of ICSOs who reported CSA exposure, 82% of those abused before age 11 targeted victims under 11, while 71% of those abused at 11 or older offended against victims in the same age group, indicating a significant association (p=0.004) between offenders’ age at the time of abuse and the age of their victims. Additionally, 86% of those who reported CSA by family members, predominantly targeted children within their households, suggesting a link between family-based CSA and intra-familial offending (86% vs. 48%, p-value= 0.059). Furthermore, among the 55% exposed to physical violence, more used physical force on their victims than those who did not report such adverse experiences (75% vs. 25%, p-value 0.401).

**Conclusions:**

The findings indicate a potential link between offenders’ own CSA experiences and their subsequent victim selection, supporting the hypothesis that reenactment of trauma may play a role in offending behavior (Garbutt et al., 2023). These results emphasize the importance of understanding the influence of early trauma to inform prevention and intervention strategies aimed at reducing CSA perpetrated by both children and adults with prior victimization histories. However, most victims of such trauma do not reenact their trauma.

**Disclosure of Interest:**

None Declared

